# Venous Thrombo-Embolism in Hospitalized SARS-CoV-2 Patients Treated with Three Different Anticoagulation Protocols: Prospective Observational Study

**DOI:** 10.3390/biology9100310

**Published:** 2020-09-24

**Authors:** Yaroslava Longhitano, Fabrizio Racca, Christian Zanza, Marina Muncinelli, Alberto Guagliano, Elisa Peretti, Anna Chiara Minerba, Marta Mari, Riccardo Boverio, Mario Salio, Guido Chichino, Francesco Franceschi, Andrea Piccioni, Ludovico Abenavoli, Mauro Salvini, Marco Artico

**Affiliations:** 1 Department of Anesthesiology and Critical Care Medicine, Azienda Ospedaliera SS. Antonio e Biagio e Cesare Arrigo, 15121 Alessandria, Italy; lon.yaro@gmail.com (Y.L.); fracca@ospedale.al.it (F.R.); 2 Department of Anesthesia, Critical Care and Emergency Medicine, Pietro and Michele Ferrero Hospital, 12051 Verduno-Alba, Italy; 3 Department of Anesthesiology and Emergency Sciences, Policlinico Gemelli/IRCCS-Catholic University of Sacred Heart, 00168 Rome, Italy; francesco.franceschi@unicatt.it (F.F.); andrea.piccioni@policlinicogemelli.it (A.P.); 4 Department of Vascular Surgery, Azienda Ospedaliera SS. Antonio e Biagio e Cesare Arrigo, 15121 Alessandria, Italy; mmuncinelli@ospedale.al.it (M.M.); aguagliano@ospedale.al.it (A.G.); elisa.peretti@libero.it (E.P.); aminerba@ospedale.al.it (A.C.M.); marta.mari.88@alice.it (M.M.); msalvini@ospedale.al.it (M.S.); 5 Department of Emergency Medicine, Azienda Ospedaliera SS. Antonio e Biagio e Cesare Arrigo, 15121 Alessandria, Italy; rboverio@ospedale.al.it; 6 Department of Internal Medicine, Azienda Ospedaliera SS. Antonio e Biagio e Cesare Arrigo, 15121 Alessandria, Italy; mario.salio@ospedale.al.it (M.S.); guido.chichino@ospedale.al.it (G.C.); 7 Department of Health Sciences, Magnae Grecia University of Catanzaro, 88100 Catanzaro, Italy; l.abenavoli@unicz.it; 8 Department of Sensory Organs, Sapienza University of Rome Policlinico Umberto I, 00155 Rome, Italy; marco.artico@uniroma1.it

**Keywords:** Covid-19, acute respiratory failure, deep vein thrombosis, coagulopathy, venous thromboembolism, thromboprophylaxis

## Abstract

**Simple Summary:**

The aims of this study are: (1) to analyze the risk of vein thrombosis and pulmonary embolism in patients affected by pneumonia due to Covid-19; (2) to evaluate conditions that could increase this risk; (3) to verify the efficacy of different doses of antithrombotic drugs to prevent these life-threatening complications. Seventy-four patients were enrolled (44 men and 30 women, average age 68.6). All of them were screened with lower limb ultrasound. Laboratory analyses including D-dimers were tested the same day. In case of clinical suspicion of pulmonary embolism, they performed a CT pulmonary angiography. A total of 28.4% (21 patients) were diagnosed with deep vein thrombosis or pulmonary embolism. This finding confirms that these patients were at increased risk of venous thromboembolism, as already reported from other studies. Mechanical ventilation, higher d-dimer levels, longer length of hospitalization and admission to intensive care unit showed to be statistically associated with thromboembolic events. In addition, the study showed that an intermediate or high dose of anticoagulation did not decrease the risk of thromboembolic events compared to lower doses. On the other hand, six patients reported severe bleeding that could be caused by higher doses of anticoagulant drugs.

**Abstract:**

The purpose of this study is to assess thrombotic risk in CoViD-19/pneumonia patients with acute respiratory failure and to compare populations treated with three different antithrombotic prophylaxis protocols. The primary outcome is to analyze the prevalence of thrombotic events in hospitalized patients, while the secondary outcome is to analyze the correlation between different anticoagulation targets with thrombotic events. All patients referred to our hospital for acute respiratory failure due to COVID-19 pneumonia between 18 and 31 May 2020 were included. Seventy-four patients were enrolled (44 men and 30 women, average age 68.6). Diagnosis of venous thromboembolism was made in 21 cases (28.4%) and thrombotic events were associated with positive pressure ventilation support (*p* = 0.024) and hospitalization in ICU (*p* < 0.0001). These patients presented higher levels of D-dimer (*p* < 0.0001) and their hospital length of stay was >16 days longer. Forty-seven out of 74 patients (63.5%) received intermediate or therapeutic dose of anticoagulation, while twenty-seven patients (34.5%) received standard antithrombotic prophylaxis. The analysis showed that an intermediate or therapeutic dose of anticoagulation did not decrease the prevalence of thrombotic events. On the other hand, six patients reported severe hemorrhagic complications. Despite intermediate or therapeutic-dose of anticoagulation, a high number of patients with acute respiratory failure secondary to COVID-19 developed thrombotic complications.

## 1. Introduction

A novel coronavirus was identified in late 2019 that rapidly reached pandemic proportions. The World Health Organization has designated the disease caused by the virus as coronavirus disease 2019 (COVID-19). Bilateral pneumonia, acute respiratory failure (ARF), systemic inflammation, endothelial dysfunction and coagulation activation have been described as key features of severe COVID-19 [1,2,3,4,5,6]. Among hospitalized COVID-19 patients, an increased risk of venous thromboembolism (VTE) has been reported despite adequate thromboprophylaxis [7,8,9,10]. Patients in the ICU had a higher risk of VTE (30.4%) than those in the ward (13.0%) [10]. Based on these reports, many physicians are advocating the empiric use of therapeutic anticoagulation even in patients who do not have a documented diagnosis of VTE [7,11,12]. On the other hand, the current position of the majority of medical societies still recommend using standard prophylactic doses of anticoagulation for hospitalized COVID-19 patients, similar to what is recommended for other acutely ill medical patients [13]. At the same time, in our general ICU, a high prevalence of pulmonary thrombo-embolism (PE) was registered among the first 62 patients (19.3% cases) affected by COVID-19-related acute respiratory failure (ARF), admitted from 1 March to 31 March 2020, despite a regular antithrombotic prophylaxis [14]. Thus, a protocol with increased doses of thromboprophylaxis was introduced in our hospital for these patients. Our goal was to estimate the burden of asymptomatic deep vein thrombosis (DVT) in subsequent patients admitted to our hospital with ARF related to COVID-19 pneumonia in relation to the antithrombotic protocol applied.

## 2. Materials and Methods

This was a prospective, observational study performed at SS. Antonio and Biagio and Cesare Arrigo—a quaternary Teaching Hospital of Alessandria. Between 18 and 30 May 2020, all consecutive patients referred for ARF due to COVID-19 pneumonia were screened for asymptomatic DVT and recruited in three medical wards and in the general ICU of Alessandria Hospital. Patients older than 18 years were included. All subjects included in the study underwent bilateral complete duplex ultrasound (CDUS) to screen for DVT. The same day, D-dimers were also tested in all patients. Covid-19 pneumonia diagnosis was made by clinical features and positive polymerase chain reaction on nasopharyngeal swab. ARF diagnosis was defined by PaO_2_/FiO_2_ < 300 or SpO_2_ < 90% in room air. In order to decrease confounding factors, patients with previous coagulative disorders, polyglobulia, anticoagulant chronic therapy, previous DVT diagnosis or diagnosis of cancer-related DVT were excluded. Patients were managed following current guidelines [15] and hypoxia was treated by O_2_ therapy, high-flow nasal cannula, noninvasive mechanical ventilation (NMV) or invasive mechanical ventilation (IMV) according to the severity of ARF. All patients received anticoagulant drugs at prophylactic, intermediate or therapeutic dose. Local protocol for standard antithrombotic prophylaxis consisted of enoxaparin 80 U/kg per day or heparin 5000 U every 8 h. Therapeutic-dose (full-dose) anticoagulation protocol included two possible options: (1) heparin 12,500 U every 8-12 h in order to obtain a PTT ratio greater than 1, 5 or (2) enoxaparin 100 U/kg every 12 h. A dose of enoxaparin or heparin between prophylactic and therapeutic dosage was classified as intermediate. As a high anticoagulation dose increases bleeding risk, the type of antithrombotic protocol was chosen by doctor after assessing the risk of thrombosis and bleeding for each patient.

The protocol of this single-center, prospective, observational study has been reviewed and approved by the local ethics committee (IRB of Alessandria n. #0011319). Participants were assured of the confidentiality and privacy of all collected information, which was recorded on a password-protected Excel database. All demographic characteristics, medical history, comorbidities, date of first symptoms onset, clinical signs, biological and imaging data were included.

### 2.1. Outcomes

Primary outcome was the occurrence of VTE.

Secondary analysis was carried out regarding VTEs site, its correlation with type and dose of antithrombotic therapy and hospital length of stay, relation between VTE and severity of ARF, type of respiratory support and principal comorbidities. The mortality at 40 days was also evaluated.

### 2.2. Laboratory Analysis and Imaging

Coagulation activity (PT, aPTT, fibrinogen, D-dimers) and platelet count were tested on the same day of CDUS. A scoring system published by the International Society on Thrombosis and Haemostasis (ISTH) in 2009 was used to evaluate the presence of disseminated intravascular coagulation (DIC) [16]. Additionally, patients underwent arterial blood gas analysis (ABG), and a routine blood test including LDH, ferritin and C-Reactive Protein (CRP).

All patients underwent at least one bilateral CDUS after hospital admission and underwent additional CDUS upon clinical suspicion of DVT. Bilateral CDUS was performed by an experienced vascular surgeon, and included the femoral-popliteal district, saphenous trunks, sapheno-femoral and sapheno-popliteal junction and calf veins to evaluate the burden of DVT [17]. Upper extremities and jugular examinations were made only on clinical suspicion of catheter-related thrombosis. A definite DVT event was recorded only if one of the following was reported: no compressibility of one or more venous segments by upper or lower limb compression ultrasound. Patients with suspected PE, based on their clinical or laboratory parameters evolution (e.g., unexplained worsening of PaO2/FIO2 ratio, unexplained hemodynamic instability, signs of acute right heart failure, marked elevation of D-dimer levels), also performed a CT pulmonary angiography (CTPA). A definite PE event was recorded only if a chest CT scan documented a segmental or more proximal intraluminal filling defect. 

### 2.3. Statistical Analysis

Continuous data were represented as mean and standard deviation or median with interquartile ranges: differences between groups were tested, depending on distribution, by Student *t*-test or Mann–Whitney U test. Categorical variables underwent Chi-squared test or Fisher’s exact. Odds ratios and their 95% confidence intervals were used to determine the strength of association between risk factors and outcomes. Multiple group comparison was made by ANOVA or Kruskal–Wallis (post hoc Mann–Whitney), as appropriate. The correlation between two variables was tested by Spearman test. Relationships between coagulative patterns and composite outcome were investigated through logistic linear regression models. A two-tailed *p* value of 0.05 was considered as significant. Statistical analysis was carried out with IBM SPSS statistics for Windows (2016), Version 24.0. Armonk, NY, USA.

## 3. Results

Seventy-four patients met the eligibility criteria and were then recruited. No missing data were detected. Demographic characteristics and principal comorbidities are summarized in Table 1. 

All recruited patients presented fever as first symptom of illness, 29% also presented cough and 68% presented dyspnea. Symptoms started, on average, 6.5 (2–11) days before hospital admission. The CDUS was performed 9 (5–18.17) and 14 (6–23.75) days after hospital admission and onset of symptoms, respectively.

Average PaO2/FiO2 was 222.26 (±59.33). Hypoxemia was treated by low-flow oxygen therapy, nasal high-flow (NHF) therapy, helmet CPAP and invasive mechanical ventilation, respectively, in 29.72%, 10.71%, 37.83% and 21.42% patients. Groups which received NHF, CPAP and invasive mechanical ventilation were unified in a positive pressure respiratory assistance group that comprised 53 patients.

### 3.1. VTE Prevalence 

Diagnosis of VTE was made in 21 cases (28.4%), in particular, nine pulmonary thromboembolism and 12 DVT. CT pulmonary angiography was executed on 34 patients, based upon clinical suspicion. Most of PE patients had thrombi in segmental branches of the pulmonary artery; multiple peripheral defects were found only in two cases. The description of venous thrombosis location is shown in Table 2. During the performance of CDUS, 11 cases of superficial vein thrombosis (SVT) were also found. SVT did not correlate with PE occurrence, while a strong association was found between PE diagnosis and DVT (risk ratio; 8.6; approximate 95% C.l.: 2.6–28; Fisher’s exact test: *p* = 0.001). 

There were no statistically significant differences in demographic characteristics and principal comorbidities between patients which developed thrombotic events and patients who did not (Table 3). PaO_2_/FiO_2_, which represents one of the respiratory disease severity index in patients with COVID-19 pneumonia, did not significantly differ between the two groups (*p* = 0.280), but individuals treated with positive pressure respiratory assistance showed a higher prevalence of VTE (*p* = 0.024). ICU patients seem to be at higher risk for developing thrombotic events (*p* < 0.0001).

The high plasma concentration of some inflammation markers like LDH and CRP seems to be related to VTE (both *p* = 0.001) (Table 3). High plasmatic D-dimer levels were strongly related to VTE in COVID-19 patients, differently from all other coagulation indicators tested. Overall hospital LOS analysis did not reach statistical significance (*p* = 0.152), but patients with VTE reported 16 more days of hospitalization compared to those without thrombotic events. There was no difference in terms of mortality between two groups.

### 3.2. Antithrombotic Prophylaxis 

**Figure 1:**
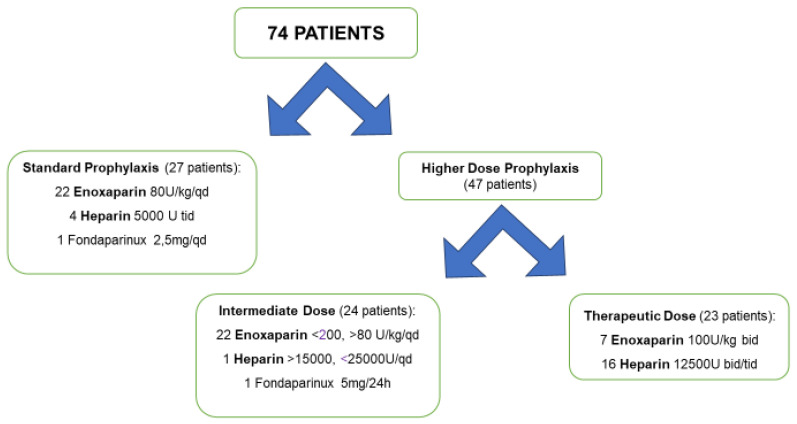
Antithrombotic prophylaxis: molecules and doses.

Prophylactic, intermediate or therapeutic anticoagulant drug doses were administered in 27, 23 and 24 cases, respectively. Specific molecules and doses are represented in Figure 1. Patients receiving intermediate and therapeutic doses were unified in higher-dose prophylaxis group, consisting of 47 patients. Comparison between these groups showed no differences in demographic characteristics, comorbidities, or baseline disease severity including PaO_2_/FiO_2_ ratio, standard laboratory tests results, CRP, LDH, D-dimer or use of positive pressure respiratory assistance (Table 4). Interestingly, antithrombotic therapy dosage was not influenced by patients’ allocation, such as ICU or general ward. This statistical model highlights that in these two statistically comparable groups, the rates of thrombotic events were not significantly different between a standard and higher dose of anticoagulant prophylaxis, neither as number of patients affected by DVT (*p* = 0.210) nor as burden of DVT (*p* = 0.412), which considers the number of vascular beds affected by the thrombosis instead of singular patients. On the other hand, six cases of major bleeding complication were registered in our cohort, two cases with standard antithrombotic prophylaxis and four cases with increased antithrombotic dose. Three cases of hemorrhagic shock, and three cases of significant spontaneous bleeding (two cases of psoas muscle hemorrhage and one case of gastrocnemius hemorrhage) were reported. Finally, mortality was three times higher in patients with increased dose of antithrombotic prophylaxis group than in standard prophylaxis subjects (OR 3.38) (Table 4), even though the statistical significance probably was not reached because of the low number of events.

## 4. Discussion

Covid-19 may predispose patients to thrombotic disease [16,18,19], due to excessive inflammation, platelet activation, and endothelial dysfunction, which is associated with a higher rate of mortality [18]. Indeed, in critically ill patients with impaired cardiopulmonary reserve, a small PE might have severe or fatal sequelae [20]. 

According to Helmes et al. [7], a systemic inflammatory response syndrome, assessed by high fibrinogen, was present in all patients and could be responsible for the activation of blood coagulation, as demonstrated by D-dimers’ elevation in almost all (>85%) patients. Other coagulation parameters reaffirmed precedent studies [7,20]: PT and aPTT are normal or slightly prolonged, and platelet count is normal; moreover, no patient was diagnosed with DIC nor met the criteria for probable DIC using ISTH score. These data are consistent with previous findings showing that coagulation abnormalities in patients with COVID-19 usually appear to be distinct from DIC [7,8], even though DIC has been reported in severely affected patients [5].

We found a higher prevalence of VTE (28, 4%) compared to non-COVID-19 ICU patients (2 to 8%) [7,21,22]. This result is consistent with the literature. In fact, case series of ICU patients including more than 600 patients reported high rates of VTE (range 20 to 43%), mostly PE [8,23,24,25]. Data regarding VTE rate outside the ICU are more limited, but also suggest a possibly increased rate (range 3% to 6%) [16,25]. Other studies focused on Covid-19 patients also show a higher rate of DVT (65–69% in ICU patients [26,27], 11.9–21% in general ward patients [9,28,29]). Unlike what has been described by other authors [9,28], in our study most DVTs were proximal (84.6%) and only 15.4% of DVTs were distal. In addition, we reported PE localized in segmental branches of the pulmonary artery in most of the patients and a strong association was found between PE and DVT, suggesting a typical thromboembolic origin. However, the filling defect in pulmonary vessels detected by CTPA could be caused by pulmonary embolism or pulmonary thrombosis [30,31]. Consequently, the prevalence of PE from the present study may still be overestimated. 

We found that there were no differences in terms of mortality between patients diagnosed with VTE and the other patients. Of note, our study showed that patients with VTE had a prolonged hospital length of stay (>16 days longer), which may be an important risk factor for DVT. 

Increased levels of CRP and LDH, due to inflammatory reaction and tissue destruction, are significantly associated with the severity of the Covid-19 disease [32]. In our study, higher concentrations of these biomarkers seem to be related to VTE. Moreover, according to the literature our data showed that VTE was significantly associated with elevated D-dimer [9,19,28], and with positive pressure respiratory assistance [28]. Findings from the present study further support the inclusion of D-dimer measurement in VTE risk assessment among acutely ill hospitalized patients with or without COVID-19. Among patients treated in medical wards, helmet CPAP was the most frequent respiratory assistance, as recommended by the literature [33]. We considered high-flow nasal cannula as positive pressure assistance because it can develop a positive pressure in the nasopharynx. In particular, Parke et al. [34] showed that 50 L per minute produced a mean pressure of 3.3 cm H_2_O in the nasopharynx with the mouth closed and 1.7 cm H_2_O with the mouth open. 

The comparison between ICU and general wards patients confirmed that COVID-19 patients admitted to ICU developed significantly more thrombotic complications. Several factors contribute to the increase in VTE risk in ICU patients. Recognized risk factors for DVT are related to one or more elements of Virchow’s triad: flow stasis, vessel injury and hypercoagulability. Flow stasis, due to prolonged immobility, mechanical ventilation, use of sedatives and neuromuscular block, plays a major role in ICU patients [35,36,37]. In addition, in this population vessel injury may be due to catheter insertion in central veins and hypercoagulability may be induced by sepsis or dehydration [35,36]

Thrombotic complications occurred in our cohort of patients with COVID-19 pneumonia despite prophylactic or increased anticoagulation targets. Indeed, 63.5% of our patients received higher anticoagulation targets. These data confirm some previous findings of ICU COVID-19 patients. In a French study, 43% of patients reported VTE despite thromboprophylaxis, and thrombotic complications occurred despite prophylactic or therapeutic anticoagulation, respectively, in 70% and 30% of patients [7]. In another series, among 74 patients, VTE was reported in 29 patients [25]. All of them were receiving anticoagulation, both at prophylactic and therapeutic levels.

Our study shows no benefit with higher doses of anticoagulant therapy in terms of thrombotic events. Moreover, mortality among patients receiving a higher dose of antithrombotic prophylaxis was three times higher than in subjects treated with standard prophylaxis. In addition, six patients in our study reported a major bleeding (two cases with standard antithrombotic prophylaxis group and four cases with increased antithrombotic dose), with a hemorrhagic shock in three cases. The mechanisms that explain this finding remain unclear. We know that heparin resistance may be a concern in acutely ill patients with COVID-19. Indeed, a series of 15 individuals treated in the ICU for VTE had a very high requirement for unfractionated heparin or low molecular weight heparin [38]. The reason for heparin resistance is not understood; the authors stated that heparin is negatively charged and can interact with a variety of positively charged plasma proteins, some of which behave like acute phase reactants and will compete for heparin binding. 

Given the limited total number of patients in our study and the non-randomized design, it may be premature to disprove the benefit of higher dose thromboprophylaxis. Furthermore, the suboptimal efficacy of higher anticoagulation dose could also be explained by the underlying pathophysiological mechanism which explains the presence of thrombotic material in pulmonary circulation [30,31]. In the context of COVID-19, pulmonary thrombosis may develop via a distinctive mechanism, and therefore may not respond adequately to intensified anticoagulation. Thus, our data confirm that the choice between different anticoagulation treatments is challenging. Hypercoagulability appears to adversely impact prognosis, but there are no high-quality studies to support interventions that go beyond standard indications, while antithrombotic therapies increase the risk of bleeding [39]. In addition, to date, there are no studies comparing different levels of anticoagulation in these patients (prophylactic, intermediate, or therapeutic dosing), and clinical trials aiming to determine the best approach for critically ill patients are in progress [40]. 

Our study has some limitations. The first and the most important limitation of our study is a limited simple size. Therefore, our findings need to be confirmed in larger samples. Secondly, CDUS was performed not at the same time-point for all patients, therefore it is possible that DVT developing at later stages during hospital stay was not identified. Finally, we looked for PE only in symptomatic patients.

## 5. Conclusions

In conclusions, our study demonstrated the occurrence of thrombotic complications in a high number of patients with ARF secondary to COVID-19 infection, irrespective of anticoagulant dosage. Further and larger studies are now needed in order to determine the best thromboprophylactic strategy in COVID-19 patients. 

## Figures and Tables

**Table 1:** Demographic characteristics and principal comorbidities of intensive care unit (ICU) and general ward patients. Parametric data were represented as mean (standard deviation) and non-parametric data as median <1st, 3rd interquartile>.

	ICU (18 Patients)	General Ward (56 Patients)	Total (74 Patients)
Gender, *n* (%)			
*Male*	15 (83.3%)	29 (51.8%)	44 (59.5%)
*Female*	3 (16.6%)	27 (48.2%)	30 (40.5%)
Age, (years)	60.22 (±10.49)	71.46 (±15.45)	68.65 (±15.12) *p* = 0.002
Comorbidities, *n* (%)			
*AF*	1 (5.5%)	2 (4.3%)	3 (4%)
*CRF*	3 (16.6%)	4 (8.7%)	7 (9.5%)
*Obesity*	4 (22.2%)	2 (4.3%)	6 (8.1%)
*DM*	5 (27.7%)	12 (26.1%)	17 (22.9%)
*Cardiovascular disease*	9 (50%)	23 (50%)	32 (43.3%)
*COPD*	2 (11.1%)	6 (13.0%)	8 (10.8%)
*Asthma*	0 (0%)	0 (0%)	0 (0%)
*Cancer*	2 (11.1%)	6 (13%)	8 (10.8%)
Days from hospital admission to CDUS	-	-	9 <5–18.7>
Days from symptoms onset to CDUS	-	-	14 <6–23.7>

Legend: ICU, intensive care unit; AF, atrial fibrillation; CRF, chronic renal failure; DM, diabetes mellitus; COPD, chronic obstructive pulmonary disease; CDUS, complete duplex ultrasound. Italics: Distinguish subgroups.

**Table 2:** Anatomical location of peripheral venous thromboses.

	Unilateral	Bilateral	Total
DVT, proximal	1	4	5
DVT, distal	2	-	2
DVT proximal + superficial, lower limbs	2	-	2
Superficial, lower limbs	4	5	9
Superficial, lower limbs + jugular	1	-	1
Superficial, upper limbs + jugular	1	-	1
Jugular, isolated	1	-	1

Legend: DVT, deep vein thrombosis.

**Table 3:** Association analysis between venous thrombotic events and principal demographic, clinical characteristics and comorbidities. Parametric data were represented as mean (standard deviation) and non-parametric data as median <1st, 3rd interquartile>.

	Venous Thrombosis Positive (21 Patients)	Venous Thrombosis Negative (53 Patients)	ODD Ratio	*p*-Value
Gender, *n* (%)				
*Male*	15 (71.4%)	29 (54.7%)	2.07 <0.71–5.98>	0.197
*Female*	6 (28.6%)	24 (45.3%)		
Age, (years)	66.7 (±16.78)	69.40 (±14.54)	-	0.529
Comorbidities, *n* (%)				
*AF*	0 (0%)	3 (5.7%)	-	0.266
*CRF*	2 (9.5%)	3 (5.7%)	1.75 <0.32–9.65>	0.551
*Obesity*	1 (4.8%)	6 (11.3%)	0.39 <0.06–2.49>	0.385
*DM*	5 (23.8%)	11 (20.7%)	1.19 <0.37–3.82>	0.774
*Cardiovascular disease*	8 (38.1%)	24 (45.3%)	0.74 <0.27–2.04>	0.574
*COPD*	2 (9.5%)	6 (11.3%)	0.82 <0.17–3.89>	0.822
*Asthma*	0 (0%)	0 (0%)	-	-
*Cancer*	1 (4.8%)	9 (16.9%)	0.24 <0.4–1.48>	0.166
PaO_2_/FiO_2_	204 <180–236>	235 <184–281>	-	0.280
Patients underwent positive pressure respiratory assistance, *n* (%)	19 (90.5%)	34 (64.1%)	5.31 <1.27–22.16>	**0.024**
Patients allocation, *n* (%)				
*ICU*	12 (57.1%)	6 (11.3%)	10.44 <3.22–33.86>	**<0.0001**
*General ward*	9 (42.8%)	47 (88.7%)		
Laboratory tests results				
*LDH*, (U/L)	779 <631–867>	481 <359–697>	-	**0.001**
*Ferritin*, (mcg/L)	1379 <644.4–1590>	623.7 <356.5–1093>	-	0.333
*CRP*, (mg/dl)	13.78 <4.2–21.6>	1.56 <0.54–6.53>	-	**0.001**
Lymphocytes, (×10/mcL^3^)	0.74 (±0.14)	0.82 (±0.29)	-	0.371
Coagulation test results				
*Fibrinogen*, (mg/dL)	645 <421.5–795.5>	505 <413–633.5>	-	0.352
*D-dimer*, (mcg/mL)	2.78 <1.42–5.73>	1.08 <0.45–1.59>	-	**<0.0001**
*aPTT*, (s)	44.72 (±15.92)	42.12 (±9.8)	-	0.458
*PT*, (s)	15.3 (±2.6)	14.3 (±2.6)	-	0.158
*PLTS*, (×10^3^/mcL)	299.6 (±145.3)	254.3 (±116.3)	-	0.171
Hospital LOS, (days)	45.7 (±15.3)	29 (±19.5)	-	0.152
Mortality	3 (14.3%)	9 (16.9%)	0.81 <0.21–3.11>	0.777

Legend: AF, atrial fibrillation; CRF, chronic renal failure; DM, diabetes mellitus; COPD, chronic obstructive pulmonary disease; ICU, intensive care unit; LDH, lactic dehydrogenase; CRP, C-reactive protein; LOS length of stay; Italics: Distinguish subgroups; Bold: Highlight statistically significant results.

**Table 4:** Association analysis between venous thrombosis prophylaxis and principal demographic, clinical characteristics and comorbidities. Parametric data were represented as mean (standard deviation) and non-parametric data as median <1st, 3rd interquartile>.

	Standard Antithrombotic Prophylaxis (27 Patients)	Higher Dose Antithrombotic Prophylaxis (47 Patients)	ODD Ratio	*p*-Value
Gender, *n*(%)				
*Male*	14 (51.8%)	30 (63.8%)	1.639 <0.636–4.220>	0.312
*Female*	13 (48.1%)	17 (36.2%)		
Age,	65.69 (±12.77)	70.33 (±16.19)	-	0.092
Comorbidities, *n* (%)				
*AF*	0 (0%)	3 (6.4%)	-	0.180
*CRF*	1 (3.7%)	4 (8.5%)	2.419 <0.358–16.33>	0.428
*Obesity*	3 (11.1%)	4 (8.5%)	0.744 <0.169–3.276>	0.713
*DM*	4 (14.8%)	12 (25.5%)	1.971 <0.596–6.525>	0.281
*Cardiovascular disease*	9 (33.3%)	23 (48.9%)	1.917 <0.73–5.034>	0.192
*COPD*	2 (7.4%)	6 (12.8%)	1.829 <0.392–8.531>	0.475
*Asthma*	0 (0%)	0 (0%)	-	-
*Cancer*	5 (18.5%)	5 (10.6%)	0.524 <0.145–1.898>	0.34
PaO_2_/FiO_2_,	255 <220–285>	214 <175–246>	-	0.063
Patients underwent positive pressure respiratory assistance, *n* (%)	22 (81.4%)	31 (65.9%)	0.44 <0.15–1.33>	0.154
Thrombotic events, *n* (%)	10 (37%)	11 (23.4%)	0.516 <0.189–1.429>	0.210
Patients allocation, *n* (%)				
*General ward*	21 (77.7%)	35 (74.5%)	1.2 <0.40–3.56>	0.749
*ICU*	6 (22.2%)	12 (25.5%)		
Laboratory test results				
*CRP*, (mg/dL)	1.81 <0.66–12.51>	3.48 <0.96–12.31>	-	0.380
*LDH*, (U/L)	631 <360.5–818.5>	371 <443.0–752.5>	-	0.623
*D-Dimer*, (mcg/mL)	0.75 <0.34–2.73>	1.44 <0.94–2.02>	-	0.203
Hospital LOS, (days)	31.7 (±24.3)	37.26 (±28.5)	-	0.479
Mortality	2 (7.4%)	10 (21.3%)	3.38 <0.78–14.67>	0.119

Legend: AF, atrial fibrillation; CRF, chronic renal failure; DM, diabetes mellitus; COPD, chronic obstructive pulmonary disease; ICU, intensive care unit; CRP, C-reactive protein; LOS length of stay; Italics: Distinguish subgroups.
